# Influence of Ultraviolet Light and Alternating Wet–Dry Environments on the Corrosion Behavior of Weathering Steels

**DOI:** 10.3390/ma17153870

**Published:** 2024-08-05

**Authors:** Ying Yang, Yubo Wang, Xinyu Du, Tianzi Lin, Han Wang, Fandi Meng, Fuhui Wang

**Affiliations:** 1 State Key Laboratory of Metal Material for Marine Equipment and Application, Anshan 114009, China; yying66@126.com (Y.Y.); wangyubo19920601@163.com (Y.W.); aglintianzi@163.com (T.L.); 2 Iron and Steel Research Institute, Anshan Iron and Steel Group, Anshan 114009, China; 3 Shenyang National Laboratory for Materials Science, Northeastern University, Wenhua Road 3-11, Shenyang 110819, China; dxy53231323@163.com (X.D.); w12632890h@163.com (H.W.); fhwang@mail.neu.edu.cn (F.W.)

**Keywords:** weathering steel, ultraviolet, alternating wet and dry conditions

## Abstract

The corrosion behaviors of two bridge steels (Q345q and Q500q) under simulated ultraviolet irradiation and a wet–dry alternating (UVWD) environment were studied. Weight loss measurement, stereomicroscope observation, SEM, XRD, and electrochemical impedance spectroscopy (EIS) were performed to investigate the influence of the coupled environment. The results revealed that the corrosion rates of Q345q and Q500q were significantly accelerated by the synergistic effect of UV light exposure and alternating wet–dry conditions. Numerous voids and cracks could be observed throughout the thickened rust layers, enabling the corrosive substances to easily penetrate through the rust layer. Q500q exhibited better corrosion resistance than Q345q due to the addition of Mo, Cr, and Ni as corrosion-resistant elements, which tended to transform the rust layer into α-FeOOH rather than γ-FeOOH during later stages of corrosion.

## 1. Introduction

Weathering steel is commonly used in bridge construction due to its superior resistance to atmospheric corrosion [1,2]. A protective layer of corrosion products can be developed on the surface of weathering steels, which helps to slow down the corrosion process. Different types of corrosion products formed in weathering steels include fine α-FeOOH, γ-FeOOH, γ-Fe_2_O_3_, and β-FeOOH under specific atmospheric conditions. These corrosion products adhere to the steel surface and prevent direct contact with the environment. The presence of alloying elements plays a crucial role in the corrosion protection of weathering steels, while environmental factors such as exposure conditions and chemical composition influence the effectiveness of this protection [3,4]. Bridge structures are constantly exposed to environmental conditions that can lead to corrosion and deterioration over time. The weathering steel used in typical marine atmospheric environments is subjected to multi-factor coupled corrosion during service [5,6,7]. The corrosion rates in different regions or countries are significantly influenced by chloride deposition, relative humidity, time of wetness, etc. [8,9,10,11,12,13]. Miura et al. believed that weathering steel is suitable in southern and central Vietnam with 0.05 mg·dm^2^/day or less of airborne salt [8]. Zhang et al. found that the difference in corrosion products is related to the humidity and Cl^−^ in the Xisha marine atmospheric environment [14]. Among the various factors that contribute to corrosion, ultraviolet (UV) light and alternating wet and dry conditions play significant roles in accelerating the deterioration process of bridge steel [15,16,17,18,19]. Understanding the effects of these factors on corrosion resistance is essential for developing effective corrosion mitigation strategies and ensuring the long-term durability of steel bridges.

Over the years, extensive research has been conducted to investigate the corrosion behavior of bridge steel and the influence of UV light and wet–dry cycles [14,20]. Wang et al. investigated the effect of UV intensity on the corrosion behavior of carbon steel exposed to a simulated Nansha atmospheric environment. They found that the roughness of the rusted sample increased and the rust layer became thicker, with many voids and cracks distributed in the rust layer after a long period of exposure. The content of FeOOH in the corrosion products was changed with UV intensity [5]. Qiu et al. explored the corrosion behavior promoted by light illumination of weathering steel in simulated marine atmospheric conditions. The results revealed that the photogenerated electron–hole pair not only facilitated anodic dissolution and chloride ion adsorption, but also led to the transformation of hematite to magnetite (Fe_3_O_4_) in the metal/corrosion layer interface [21]. Overall, UV light affects the corrosion behavior of weathering steel through the photovoltaic effect of corrosion products with semiconductor properties in simulated marine atmospheric environments. The photoelectrons and photo-vacancies participate in the redox reaction between the substrate and the atmospheric environment. The corrosion rate of steel increases significantly with UV, leading to the formation of thick rust layers with voids and cracks, especially in the early stages [15,19,22].

Regarding the effect of dry and wet alternations, most of the studies were carried out in simulated environments. Some researchers think that the results of cyclic dry and wet conditions are comparable to those of outdoor tests in terms of structure and morphology of the corrosion products [23,24]. Raphael F. et al. conducted cyclic corrosion tests of weathering steels for 6, 15, 30, 45, and 50 cycles. The spinel phase (Fe_3_O_4_/γ-Fe_2_O_3_) was the major component of the rust formed in cyclic dry and wet conditions [24]. The content of spinel increased with time exposure, leading to an increase in the corrosion rate of the steel. Wang et al. investigated the marine atmosphere corrosion behavior of a low-alloy steel with different nickel contents in alternate wet–dry conditions. They found that the addition of nickel led to an improvement in the corrosion resistance of the steel, promoting the formation of a homogeneous and compact inner rust layer. Moreover, higher nickel content in the steel accelerated the generation of a protective rust layer [25]. Alternate wet–dry conditions contribute to the corrosion process in weathering steel through factors such as oxygen content, pH changes, alloy composition, exposure to chloride ions, and moisture levels [26,27]. To summarize, the studies above have investigated the individual effects of UV light or wet–dry cycles on the corrosion behavior of bridge steel, and have shed light on the underlying mechanisms and provided valuable insights into the corrosion resistance of weathering steel. However, the understanding of the combined effects of UV light and wet–dry cycles on the corrosion resistance of weathering steel remains limited.

Therefore, this study aims to bridge this gap by investigating the synergistic effects of UV light exposure and alternating wet–dry conditions on the corrosion behavior of weathering steel. In this work, accelerated corrosion tests of weathering steel, Q500q, were conducted under controlled UV exposure and wet–dry cycles. An ordinary bridge steel, Q345q, was tested as a control group. The corrosion behaviors of the two steels under ultraviolet irradiation and a wet–dry alternating environment (UVWD), a single ultraviolet environment (UV), and a single wet–dry alternating environment (WD) were investigated. The specific parameters of the UV irradiation and condensation steps of the experiment are based on Cycle 1 provided in ASTM G154 [28], and are controlled by the ultraviolet aging system. On this basis, the dark time is used to simulate the dry environment. The corrosion rate, surface morphology, and EIS were evaluated to analyze the differences in the corrosion behavior of the two steels, which can be attributed to environmental influences and their distinct chemical compositions. The findings of this study will contribute to a better understanding of the corrosion resistance of weathering steel in bridge structures and inform the development of corrosion mitigation strategies.

## 2. Materials and Methods

### 2.1. Materials and Sample Preparation

The bridge steel samples (Q345q and Q500q in tempered state, self-produced) were cut into 50 mm × 50 mm × 4 mm for weight loss tests. The analyzed chemical compositions of the steels are provided in Table 1. Samples were polished with 120-grit, 240-grit, and 600-grit abrasive papers in turn to remove the oxide layer on the surface. The surface of the samples was cleaned with acetone and alcohol. Then, the samples were dried, weighed, and then placed in desiccators for the other experiments. The non-working surfaces of the samples were firstly wrapped by PTFE seal tape, and then sealed by rosin and paraffin with a volume ratio of 1:1. After serving in the different conditions, the paraffin attached to the tape could be easily stripped manually. Upon inspection, there were no residues on the surfaces of the samples after stripping. The weightless samples were soaked in a rust removal solution (500 mL, 38 wt.% HCl, 3.5 g C_6_H_12_N_4_, and 500 mL H_2_O) at room temperature. The results were compensated according to ISO 8407 [29]. After the complete removal of the corrosion products, the samples were cleaned with alcohol, dried and weighed. Three replications were obtained for each test and the averages were calculated. For the electrochemical measurements, one surface and four sides of the steel sheets (10 mm × 10 mm) were sealed in rosin and paraffin before the corrosion experiments. Finally, the samples were dried in an oven at 60 °C for 5 min and kept in a dry vessel. The samples with dimensions of 20 mm × 20 mm × 4 mm were used to observe morphologies and analyze corrosion products.

### 2.2. Cyclic Corrosion Acceleration Tests

**Figure 1:**
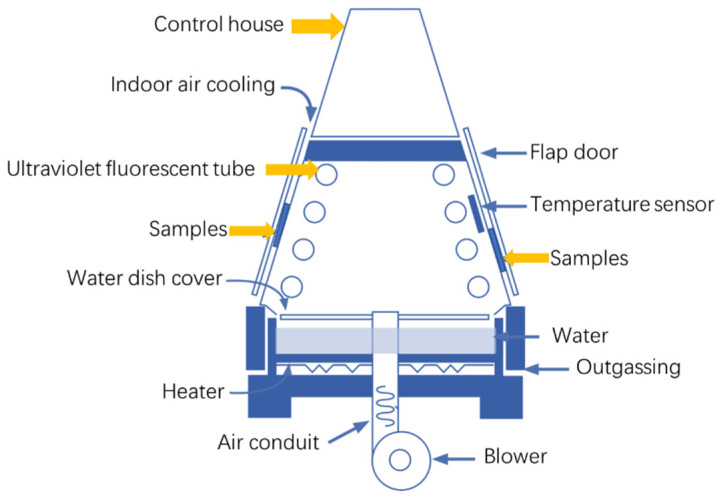
Schematic diagram of the accelerated UV aging chamber.

Three simulated corrosion environments were involved in this study: UVWD, UV, and WD. The UVWD and UV simulations were conducted using an accelerated UV aging machine (QUV-90, Q-Lab Instruments, Westlake, OH, USA) with a UV lamp (UVA-340, Q-Lab Instruments, USA). A schematic diagram of test chamber is shown in Figure 1. The specific parameters for the cycling steps in the experiments were based on Cycle 1 provided in the ASTM G154 standard [28]. For simulating the UV irradiation and wet–dry alternating conditions, the cycling steps and specific parameters were set as follows: (1) UV at 60 °C for 8 h, 0.89 W/m^2^, (2) condensation at 50 °C for 4 h, and (3) in the dark at 50 °C for 12 h. This cycle was repeated for 14 days. Dark periods were added during the cycle to simulate the dry environment. For simulating single UV conditions, the cycling steps and specific parameters were as follows: (1) UV at 60 °C for 8 h 0.89 W/m^2^, and (2) in the dark at 50 °C for 16 h. This cycle was also repeated for 14 days. For simulating the WD environment, a wet heat chamber (LRHS-1000-LH, Linpin Instruments, Shanghai, China) was used. Distilled water was used as the wetting solution, which was consistent with the solution in the UVWD process. The cycling steps and specific parameters were as follows: (1) 60 °C at 50%RH for 8 h, (2) 50 °C at 80%RH for 4 h, and (3) 50 °C at 50%RH for 12 h. This cycle was also repeated for 14 days. Analyses were conducted on the samples after subjecting the samples to 14 days of corrosion under each of the three conditions.

### 2.3. Characterization of the Rust Layers

The surface state of the samples after service was observed by a stereomicroscope and scanning electron microscopy (SEM, JSM-7001F, JEOL, Tokyo, Japan). The corresponding elemental compositions and distribution were determined using equipped energy disperse spectroscopy (EDS). Afterwards, the rust layers were scraped with a knife to obtain the dried rust powders, which were analyzed by X-ray diffraction (XRD, SmartLab, Rigaku, Japan). A Bruker D8 XRD analyzer with a Co target and a Bruker D2 XRD analyzer with a Cu target were applied to perform the measurements. The scanning angle ranged from 5° to 45° with a step rate of 4°/min. The software Jade 6.0 was used to analyze the spectra.

### 2.4. Electrochemical Test

The electrochemical impedance spectrum (EIS) measurements were performed with the traditional three-electrode system in 0.1 mol/L Na_2_SO_4_ solution, which is stable enough and has minimal effect on bare and rusty steel when testing the sample [8,20,24]. Due to the minimal effect of Na_2_SO_4_ solution and a short test period, the solution was not de-aerated before the test. A saturated calomel electrode (SCE) was used as the reference electrode (RE), platinum foil was used as the counter electrode (CE), and the specimen to be measured was the working electrode (WE). A Princeton electrochemical workstation (PARSTAT4000A, Ametek, Berwyn, IL, USA) was used to conduct the measurements. The EIS measurement began right after stabilizing the OCP circuits each time. The stimulus signal was a sinusoidal wave with frequency range from 10^−2^ Hz to 10^5^ Hz and at an amplitude of 10 mV.

## 3. Results and Discussion

### 3.1. Corrosion Rate Analysis

**Figure 2:**
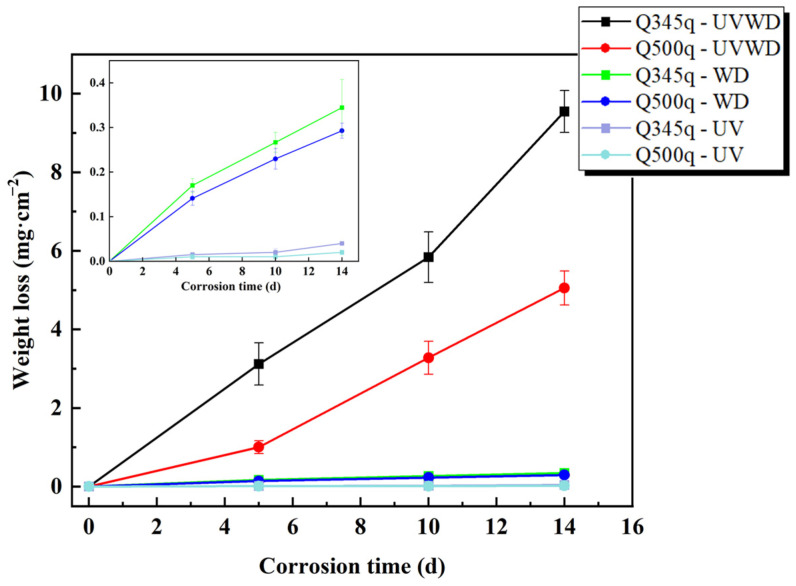
Weight loss results of the Q345q and Q500q specimens in different conditions.

**Figure 3:**
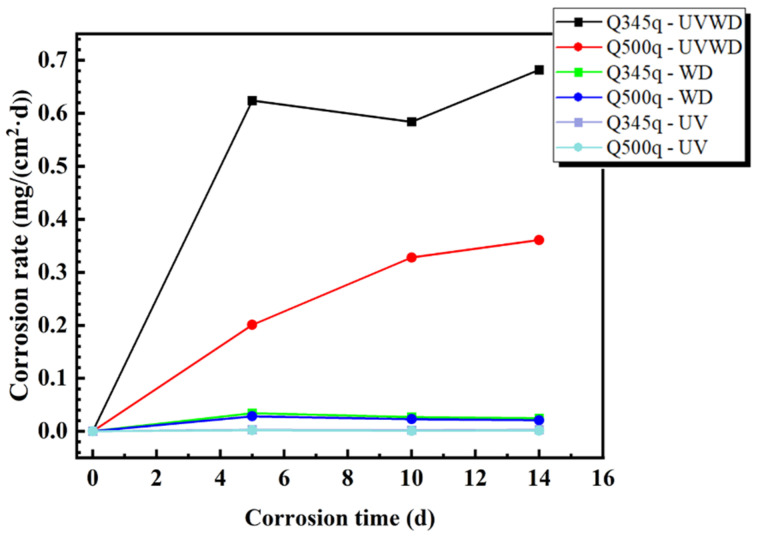
Corrosion rate results of the Q345q and Q500q specimens in different conditions.

The mass of the sample before and after corrosion under different corrosion conditions was measured by a microbalance (MC5, Sartorius, Göttingen, Germany). The mass loss rates under different corrosion periods can be quantitatively calculated using the following equation:

(1) $$w_{t}=\frac{w_{0}-w}{S}$$

where w*_t_* is the mass loss rate at corrosion time *t* (mg/cm^2^), w_0_ is the initial mass of the sample (mg), w is the mass of the sample after rust removal (mg), and S is the exposed area (cm^2^). Figure 2 and Figure 3 are the weight loss rates and corrosion rates of two steels in three simulated corrosion conditions, respectively. In Figure 2, the weight loss rates of the two specimens in UVWD conditions increase obviously as a function of the corrosion time. After servicing for 14 days, the values of the weight loss for Q345q and Q500q are 9.55 mg·cm^−2^ and 5.05 mg·cm^−2^, respectively. Compared to Q500q, Q345q shows a higher weight loss in the whole corrosion process, suggesting that the corrosion of Q345q was more severe with the increase in experimental time, and Q345q exhibits a higher susceptibility to corrosion from the beginning of the experiment. Meanwhile, it can be seen that the corrosion rates of Q345q and Q500q also increased with corrosion time (Figure 3), and that the growth in the corrosion rate of Q500q showed a slowing trend.

For the WD condition (see Figure 2), the weight loss rates of two steels increased gradually, which significantly decreased with a difference of one order of magnitude compared to the UVWD environments. The values of Q345q and Q500q after serving for 14 d were 0.34 mg·cm^−2^ and 0.29 mg·cm^−2^, respectively. The weight loss rate of Q345q was still higher than that of Q500q and in the WD environment. Although, after 5 d, the corrosion rates of two steels decreased slightly (see Figure 3), the change tendencies of the two steels were not the same as in the UVWD condition, suggest that the corrosion mechanism may have changed.

Compared to the above two conditions, the corrosion rates of the two steels in the single UV condition were almost invisible (see Figure 2 and Figure 3), which indicate that the effect of single ultraviolet irradiation on metal corrosion is relatively minor. From the analysis above, the corrosion rates of both steels were greatly enlarged under the synergistic effect of ultraviolet irradiation and alternating wet–dry environments. Since the single UV condition almost caused no corrosion and the single WD condition also caused a relatively slow corrosion rate, the corrosion mechanism in the UVWD condition may have changed.

It seems that the data in UVWD and WD conditions have slight nonlinear trends (see Figure 2). Therefore, the corrosion kinetics of the two steels in each environment were described using empirical Formula (2), which is commonly used for fitting the experiment data in the analysis of the weight loss of steels [5]. The fitting results are shown in Table 2. The kinetics fitting formula is as follows:

w = A*t*^n^ (2)

where A and n are constants obtained by the fitting results. The correlation coefficient R^2^ of UVWD and WD conditions is close to 1, suggesting that the fitting results are very good. Due to the influence of the small values under UV conditions, the determination coefficients of the nonlinear fitting for UV conditions are acceptable. The values of A and n in the UVWD and WD environments are shown in Table 2. The value of n is generally considered as a parameter reflecting the characteristics of the rust layer. The fitting values of n for Q345q and Q500q in the UVWD environments are both greater than 1, indicating that the corrosion rates of carbon steel increase with the prolongation of corrosion time, and are consistent with the trend in Figure 3. Furthermore, it can be inferred that the corrosion products fail to provide effective protection to the substrate. The A value of Q345q is higher than that of Q500q, indicating that the corrosion rate of Q345q is higher than that of Q500q in the UVWD environment from the perspective of weight loss. On the other hand, in the WD environment, the values of A show a similar trend in the corrosion rates of the two steels. The fitting values of n for Q345q and Q500q in the WD environments are both smaller than 1, indicating that the corrosion rates of carbon steel decrease with the corrosion time, which also can be found in Figure 3. As for the fitting results in the UV environments, although the n values of Q345q and Q500q are greater than and less than 1, respectively, the corrosion rates are too small to make this numerical difference meaningful.

### 3.2. Macroscopic Corrosion Morphology

**Figure 4:**
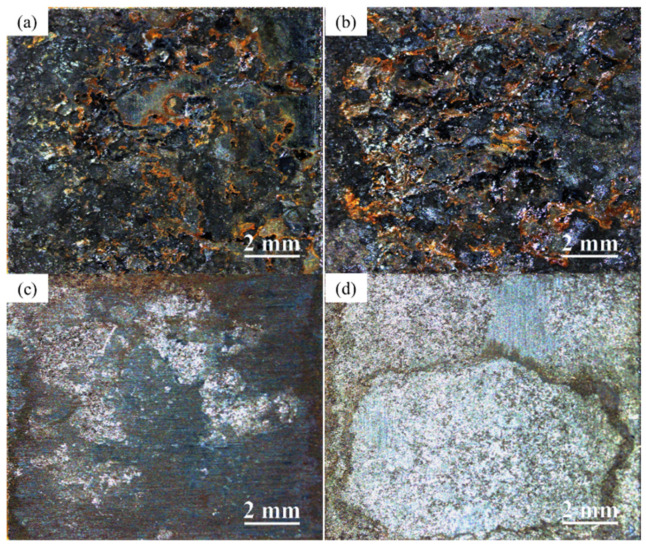
Macrophotographs of (**a**) Q345q and (**b**) Q500q in the WD condition for 14 d, and (**c**,**d**) the specimens after rust removal.

**Figure 5:**
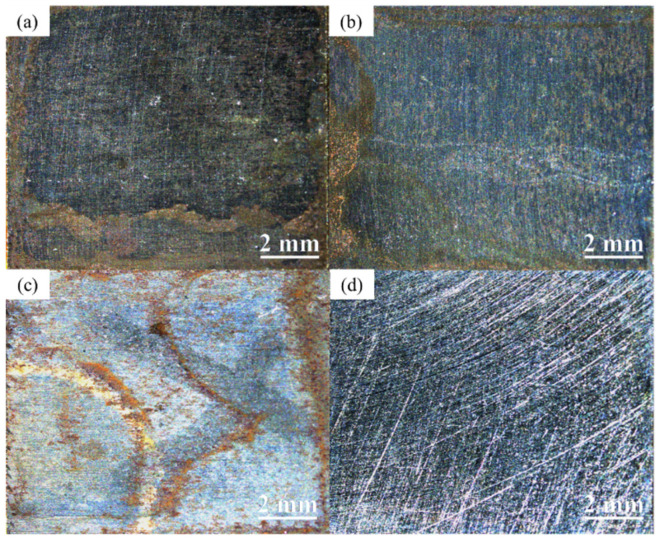
Macrophotographs of (**a**) Q345q and (**b**) Q500q in the UV condition for 14 d, and (**c**,**d**) the specimens after rust removal.

**Figure 6:**
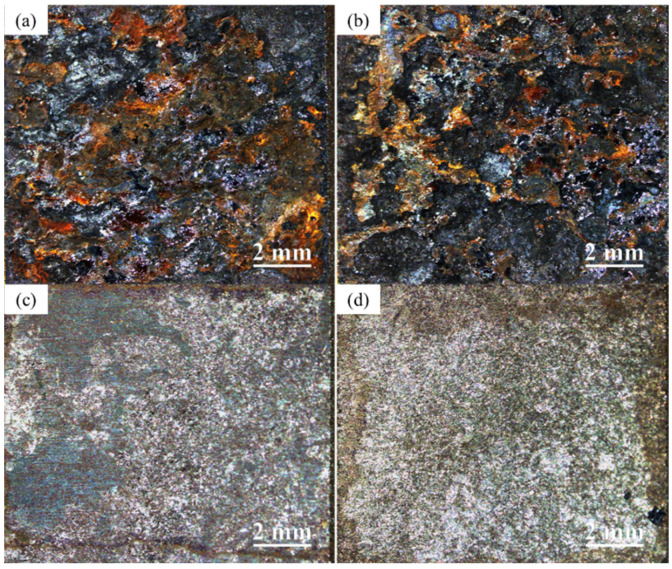
Macrophotographs of (**a**) Q345q and (**b**) Q500q in the UVWD condition for 14 d, and (**c**,**d**) the specimens after rust removal.

The surfaces of the two types of weathering steels were observed after serving for 14 d in different conditions, using a Stemi 508 stereomicroscope, as depicted in Figure 4, Figure 5 and Figure 6.

As shown in Figure 4, the corrosion levels of the two types of steel in the WD condition are similar. The difference in rust between Q345q and Q500q seems to be negligible. However, after removing rust from the surface, there was an evident difference in the proportion of corrosion pits (Figure 4c,d). A comparison between both steels reveals that Q345q corrodes significantly faster than Q500q, since the surface of Q500q appears smoother.

Regarding the macrophotographs in the single UV condition, from Figure 5 it can be seen that both steels experienced slight corrosion. The steel surface was scarcely covered by a complete film of corrosion products (Figure 5a,b). In particular, Q500q presented a highly polished appearance after rust removal. The level of corrosion was greatly reduced compared to the simulated WD condition. There were almost no signs of visible corrosion pits on the surface, indicating that the impact of the single UV condition on the corrosion of weathering steel is minimal.

It can be observed that Q345q exhibited black and reddish-brown rust formation after 14 d with the emergence of a loose rust layer in the UVWD condition (Figure 6a). After removing rust from the surface, a decrease in the smoothness of the steel matrix was noted and an increase in corrosion pits can be observed (Figure 6c). In contrast, the proportion of reddish-brown color of Q500q is less than that of Q345q (Figure 6b). An increased proportion of reddish-brown rust can be found in Figure 6a,b compared to the WD environment. A comparison between the two steels indicates that Q345q exhibits a significantly higher corrosion rate than Q500q, and the corrosion levels of the steels are significantly higher than those in the WD condition. This suggests that the loose layers of corrosion products do not provide effective protection for the substrate in the UVWD condition.

### 3.3. Microscopic Corrosion Morphology

**Figure 7:**
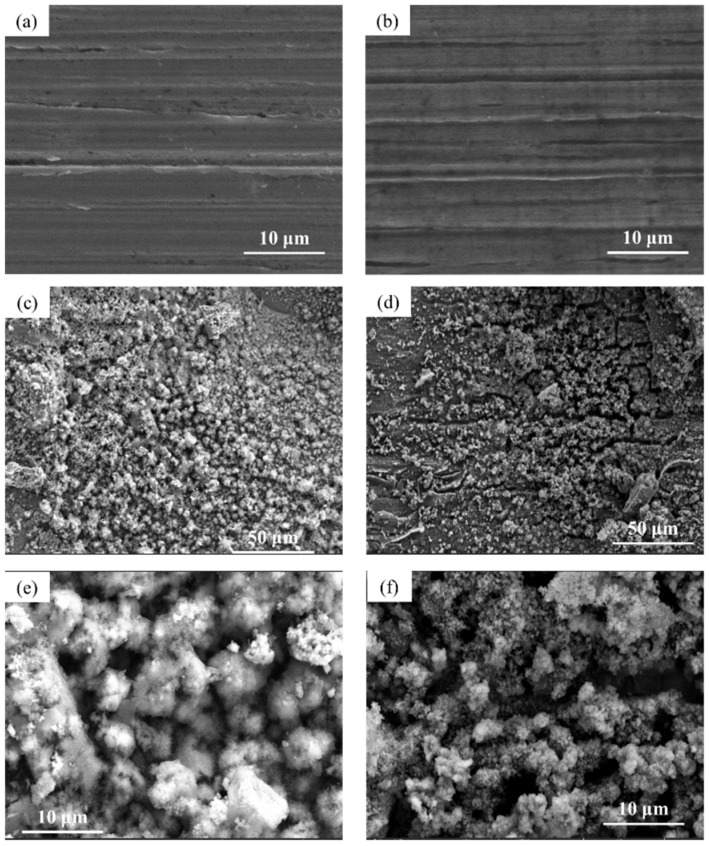
Microscopic surface morphology of (**a**) Q345q and (**b**) Q500q before testing; (**c**) Q345q and (**d**) Q500q in the UVWD condition for 14 d, and the corresponding enlarged images of (**e**) Q345q and (**f**) Q500q.

**Figure 8:**
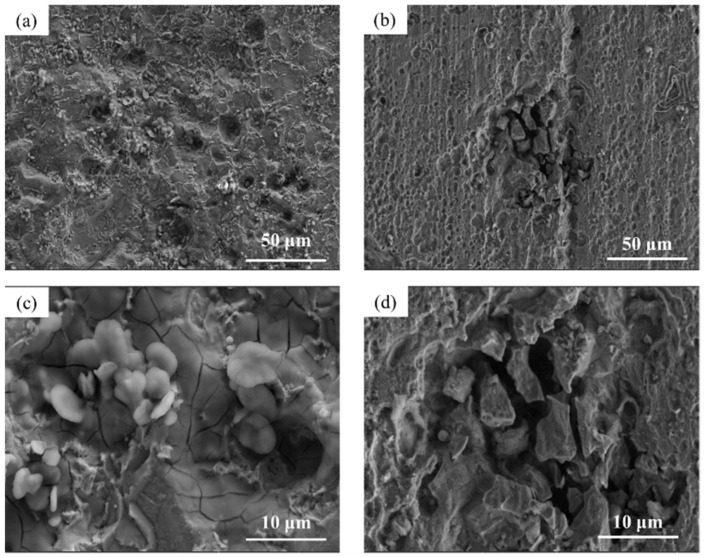
Microphotographs of (**a**) Q345q and (**b**) Q500q in the UVWD condition after rust removal, and the corresponding enlarged images of (**c**) Q345q and (**d**) Q500q.

The microscopic surface morphology of Q345q and Q500q before and after testing in different conditions was observed. In Figure 7a,b, there is very little difference in surface morphology between Q345q and Q500q. Only scratches from the sandpaper can be seen in the photos. In contrast, in Figure 7c,d, it can be observed that rust particles on the surface of the steel substrate appear to be accumulated after servicing for 14 d in the UVWD condition. The entire surfaces of the rust layers exhibit characteristics of looseness. In addition, loose clusters of particles can be observed in Figure 7e,f. There was no significant difference in the corrosion morphology of the two steels. Compared to Q345q, it seems to be that Q500q had smaller rust particles. After rust removal (Figure 8), the surface of Q345q showed a lot of pits. The surface of Q500q also exhibited some pits, while the surrounding area of these pits appeared relatively flat. The overall pitting degree of Q500q is not as serious as that of Q345.

**Figure 9:**
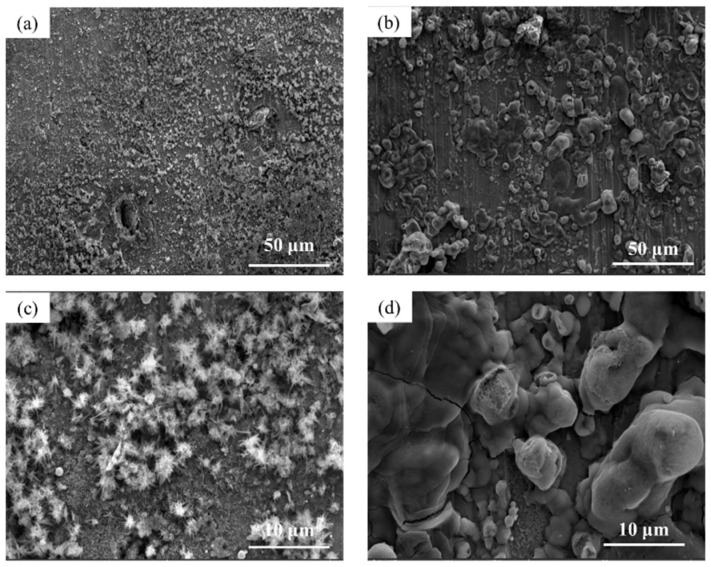
Microscopic surface morphology of (**a**) Q345q and (**b**) Q500q in the WD condition for 14 d, and the corresponding enlarged images of (**c**) Q345q and (**d**) Q500q.

Figure 9 shows the microscopic surface morphology of Q345q and Q500q in the WD condition. By comparing Figure 9a and Figure 9b, it can be observed that there is a dense layer underneath the loose particles. The surface microstructure of the rust layer on Q345q appears as scattered needle-like structures arranged densely (Figure 9c). On the other hand, the rust layer formed on Q500q exhibits a clustered surface morphology, providing better protection for the substrate. Therefore, under the single WD condition, Q500q demonstrates stronger corrosion resistance than Q345q.

**Figure 10:**
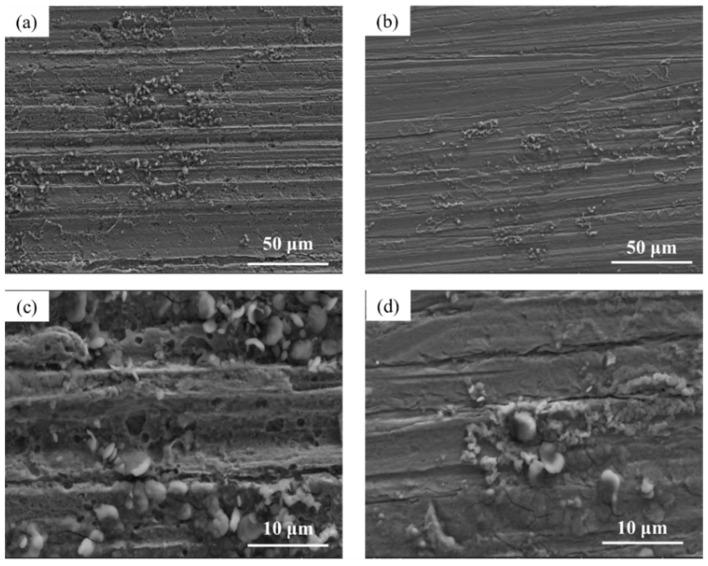
Microscopic surface morphology of (**a**) Q345q and (**b**) Q500q in the UV condition for 14 d, and the corresponding enlarged images of (**c**) Q345q and (**d**) Q500q.

The microscopic surface morphology of two steels in single UV condition after 14 days of service is shown in Figure 10. From Figure 10a,b, it can be seen that both types of steel exhibited extremely minor corrosion under UV condition. The surface still exhibits indications of the bare metal undergoing processing. In the comparison of the corresponding enlarged images, pitting corrosion was observed on Q345q, but the depth of pitting was not significant. On the other hand, no obvious signs of corrosion were found on Q500q. In any case, the corrosion of both steels in this environment is extremely weak.

### 3.4. Cross-Section Observation of the Rust Layer

**Figure 11:**
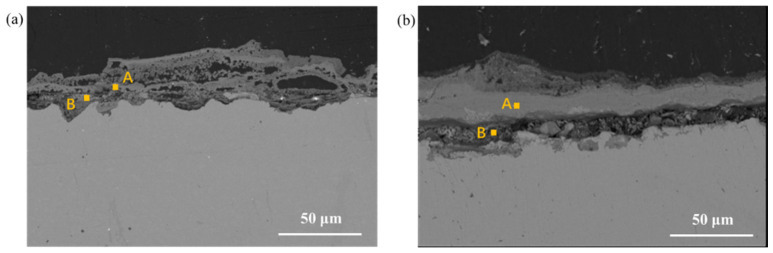
Microscopic cross-section morphology of (**a**) Q345q and (**b**) Q500q in the UVWD condition for 14 d.

**Figure 12:**
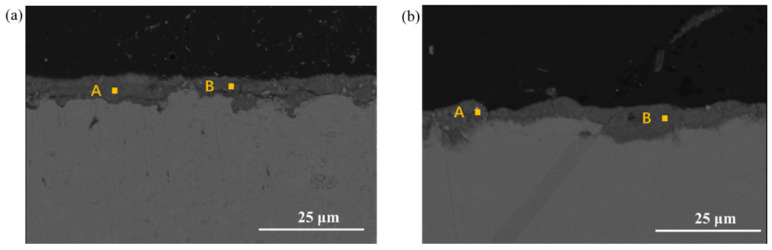
Microscopic cross-section morphology of (**a**) Q345q and (**b**) Q500q in the WD condition for 14 d.

The samples of Q345q and Q500q were subjected to 14 days of service in UVWD and WD environments, respectively, and then the cross-section observation and EDS tests were conducted, as shown in Figure 11 and Figure 12 and Table 3 and Table 4. In Figure 11, an obvious layering phenomenon is revealed in the rust layer of Q345q. The rust layer structure is relatively loose with a thin and discontinuous dense layer. According to the EDS results of the outer layer (Position A) in Table 3, the rust layer has an extremely high oxygen content with Fe and O as its main elemental composition. The iron and oxygen contents are 77.1 wt.% and 22.9 wt.%, respectively. There was little change in relative oxygen content in Position B, suggesting that the oxygen diffuses rapidly and the corrosion product layer does not have significant protective effects. The SEM image and EDS test results in Figure 11b reveal that the cross-section of Q500q exhibits a denser corrosion product layer and porous layers compared to Q345q, which forms a porous–dense–porous structure. EDS analysis indicates that the oxygen content within the compact layer is higher than that observed in the porous layer. The lower oxygen content in the inner layer could be due to its limited direct contact with the external environment. External oxygen cannot penetrate extensively, resulting in weaker oxidation, and thus the oxygen content is relatively low. Nevertheless, numerous voids and cracks can be observed throughout the rust layer in Q500q. As we know, corrosive substances can easily penetrate through the rust layer without significant hindrance from this protective barrier when large voids are present, suggesting that the rust layer formed in this environment cannot provide good protection.

Figure 12 show the cross-section morphology in the WD condition. It can be observed that the rust layer thickness of both steels is less than that in the UVWD environment. Additionally, the stratification phenomenon of the rust layer is not very pronounced, indicating that the corrosion is not serious. Further analysis using EDS confirms a relatively uniform distribution of rust layer composition. From the analysis above, it can be inferred that the UVWD condition changes the structure of the rust layer, which creates more voids and loose corrosion products.

### 3.5. Composition of the Rust Layer

**Figure 13:**
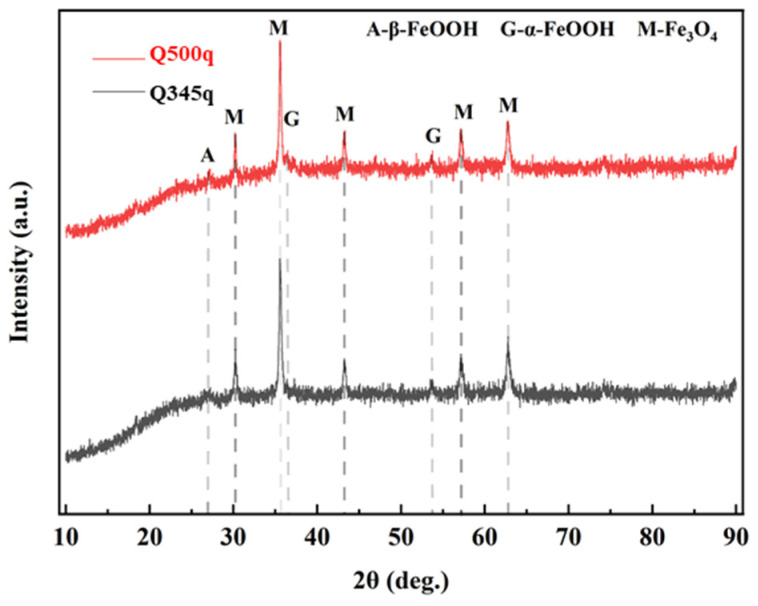
XRD spectra of corrosion products of Q345q and Q500q under the UVWD condition.

XRD testing was employed to provide further qualitative analysis of the corrosion products formed on the steels after 14 d of servicing in the UVWD condition, to elucidate the composition of the corrosion products. The XRD results are shown in Figure 13, and indicate that corrosion products of both Q345q and Q500q are mainly composed of Fe_3_O_4_, with small amounts of α-FeOOH and β-FeOOH also present. Furthermore, the difference in composition between these two types of corrosion products is not significant. The Fe_3_O_4_ and α-FeOOH in the rust layer do not provide good protective performance for the steel, which may be related to the loose structure of the rust layer formed under the synergistic effect of UV light exposure and alternating wet–dry conditions.

### 3.6. EIS Measurements

**Figure 14:**
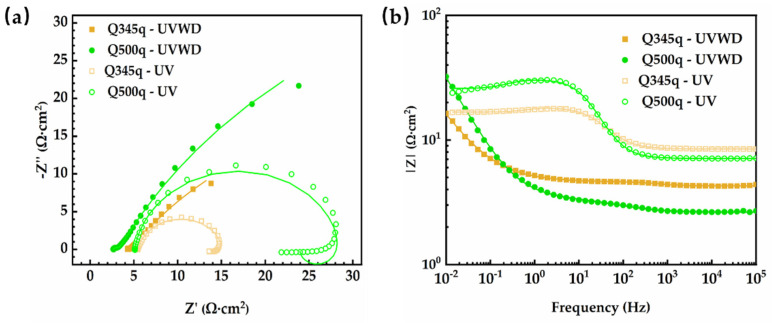
(**a**) Nyquist plots and (**b**) impedance modulus of Bode plots of Q345q and Q500q under UVWD and UV conditions.

To further investigate the protective performance of rust layers on the two weathering steels under the UVWD condition, EIS tests were conducted on both steels after 14 d of corrosion. Given that the two types of steel have been confirmed to produce negligible corrosion products under the UV exposure condition, we also performed EIS tests on both types of steel after 14 days of exposure under the UV condition (the samples were immersed in NaCl solution for EIS tests) to assess the corrosion resistance differences between the steel substrates. The Nyquist plots for the steels after 14 d of corrosion are shown in Figure 14a. It can be seen that the Nyquist plots almost reveal one capacitive characteristic, and the impedance values only reach 16 Ω·cm^2^ and 32 Ω·cm^2^, respectively, meaning that rust layers formed under the UVWD condition cannot provide enough protective performance against the corrosion. Meanwhile, two conclusions can be drawn by comparing the impedance under different conditions. First, compared to the UV condition without the formation of corrosion products, the impedance modulus of both types of steel has a very limited increase, with no order of magnitude change, further verifying that the corrosion products did not provide effective protective capacity. Second, under UV conditions, the base metal of Q500q shows a stronger trend of corrosion resistance compared to Q345q, which might be related to the addition of alloying elements.

From the analysis above, it can be inferred that UV light exposure and alternating wet–dry conditions can synergistically promote the rapid formation of corrosion products of the steels. The structures of the corrosion product layer were changed by the coupled environment. Since the impedance modulus of the steel in the UVWD condition is higher than that in the UV condition, it can be inferred that UV accelerates the corrosion of the steel, but the rust layer is not dense and does not have enough protection from the macro- and micro-morphology. Photocatalytic reactions are one of the primary mechanisms by which ultraviolet light disrupts the protective layer of corrosion products on weathering steel [5,15]. When ultraviolet light irradiates the surface of the formed corrosion products (such as Fe_2_O_3_ and Fe_3_O_4_), the energy of the photons can excite electrons within the corrosion products:

Fe_2_O_3_ + hv → e^−^ + h^+^ (3)

where photons (hv) generate electrons (e⁻) and holes (h⁺) on the surface of the products. Furthermore, the generated photogenerated electrons and holes possess high energy and can participate in further redox reactions [21]. Because the redox reactions involve photogenerated electrons and holes, the composition within the corrosion product layer undergoes continuous changes. These ongoing redox reactions can disrupt the crystalline structure of the corrosion product layer, making it more porous and brittle, and thereby causing it to lose its original density and protective properties. Therefore, the protective layer of weathering steel cannot be well formed in the UVWD condition.

Comparable porous–dense–porous layers composed of Fe_3_O_4_, with small amounts of α-FeOOH and β-FeOOH, were obtained for Q345q and Q500q. Due to the different elemental compositions, Q500q, with higher contents of Cu, Cr, and Ni, significantly exhibits a better corrosion resistance than Q345q. The addition of Cu, Cr, and Ni as corrosion-resistant elements tends to transform the rust layer into α-FeOOH rather than γ-FeOOH during the later stages of corrosion. However, the photovoltaic effect of corrosion products from UV light exposure generally serves as the primary catalyst for phase conversion [5], which in turn can manifest as blistering and fracturing on the surface of the rust layer, thereby accelerating the corrosion process. When the Ni content in the rust layer is more than 3%, it enables the inner layer of the rust to selectively exclude chloride ions, thus promoting the generation of a physical barrier against corrosion [30].

## 4. Conclusions

In summary, the corrosion rates of Q345q and Q500q were significantly accelerated by the synergistic effect of UV light exposure and alternating wet–dry conditions. UV accelerates the corrosion of the steel serving in the WD condition through photocatalytic reactions, but the rust layer is not dense and does not provide enough protection in the UVWD condition, even for the weathering steel. Because the redox reactions involve photogenerated electrons and holes, the crystalline structure of the corrosion product layer is disrupted. Numerous voids and cracks can be observed throughout the thickened rust layer, causing the corrosive substances to easily penetrate through the rust layer and the layer to lose its original density and protective properties. Q500q exhibits a better corrosion resistance than Q345q due to the addition of Cu, Cr, and Ni as corrosion-resistant elements, which tend to transform the rust layer into α-FeOOH rather than γ-FeOOH during the later stages of corrosion.

## Figures and Tables

**Table 1:** Analyzed chemical composition contents of the bridge steels (wt.%).

Element	C	Mn	Nb	V	Ti	Si	Cr
Q345q	0.13	1.55	0.01	—	0.012	0.42	—
Q500q	0.09	1.40	0.02	0.06	0.027	0.45	0.32
**Element**	**Ni**	**Cu**	**Mo**	**N**	**P**	**S**	
Q345q	—	—	—	0.004	0.015	0.002	
Q500q	0.42	0.50	0.015	0.003	0.012	0.002	

**Table 2:** Relevant parameters obtained by fitting the curves in Figure 2.

	A	n	R^2^
Q345q in UVWD	0.4204	1.1751	0.986
Q500q in UVWD	0.1085	1.4602	0.994
Q345q in WD	0.0582	0.6645	0.997
Q500q in WD	0.0450	0.7088	0.998
Q345q in UV	0.0017	1.1859	0.862
Q500q in UV	0.0213	0.8105	0.663

**Table 3:** EDS results of different positions for the two steels in Figure 11.

Sample	Position	Fe (wt.%)	O (wt.%)	Mn (wt.%)
Q345q	A	77.1	22.9	
	B	75.4	23.8	0.80
Q500q	A	74.0	26.0	-
	B	94.7	3.9	1.3

**Table 4:** EDS results of different positions for the two steels in Figure 12.

Sample	Position	Fe (wt.%)	O (wt.%)	Mn (wt.%)
Q345q	A	79.0	21.0	-
	B	78.3	21.7	-
Q500q	A	79.8	19.0	1.2
	B	80.3	18.6	1.1

## Data Availability

Data will be made available on request.
